# Conflicts of Interest and Industry Influence in Randomized Controlled Trials in Hand Surgery: A Systematic Review

**DOI:** 10.1055/s-0046-1824732

**Published:** 2026-07-28

**Authors:** Gabriel Henrique dos Santos Guimarães, Ramon Sampaio Souza Santos, Luís Renato Nakachima, Deivid Ramos dos Santos, João Carlos Belloti, Vinícius Ynoe de Moraes

**Affiliations:** 1 Department of Orthopedics and Traumatology, Escola Paulista de Medicina, Universidade Federal de São Paulo, São Paulo, SP, Brazil; 2 Department of Orthopedics and Traumatology, Orthopedic Surgery Residency Program, Universidade do Estado do Pará, Belém, PA, Brazil

**Keywords:** capital financing, conflict of interest, hand/surgery, industry, orthopedics, systematic review, conflito de interesses, financiamento de capital, indústria, mão/cirurgia, ortopedia, revisão sistemática

## Abstract

**Objective:**

To verify the association between the presence of conflict of interests and pro-funder results in randomized controlled trials (RCTs) in hand surgery. The secondary objective was to evaluate the influence of industry funding on the bibliometric characteristics of these RCTs.

**Methods:**

We examined 178 RCTs published between 2013 and 2023 in 5 high-impact journals focusing on the hand, wrist, elbow, or peripheral nerves in the MEDLINE, Embase, and Scopus databases. From the studies included, we assessed the presence of a declared conflict of interests and analyzed its association with the primary outcome (favorable vs. unfavorable). We additionally evaluated the association with other variables, including compliance with the Consolidated Standards of Reporting Trials (CONSORT) guidelines, the number of authors, and journal impact factors. The risk of bias in the included studies was assessed using the Risk of Bias 2 (RoB 2) tool. All assessments were performed by two independent reviewers, following a previously defined protocol. This systematic review was not previously recorded on public platforms such as International Prospective Register of Systematic Reviews (PROSPERO).

**Results:**

We found no relationship between the presence of conflict of interests and pro-funder results (35.4% vs. 65.6%. *p* = 0.503). Industry-funded studies have been published in journals with significantly higher impact factors than non-industry-funded studies (3.05 vs. 2.2) ( *p* < 0.001), with a significant difference in the number of funded authors (1.84 vs. 0.07) ( *p* < 0.001).

**Conclusion:**

There was no significant association between the type of funding and the achievement of favorable results in randomized clinical trials in hand surgery. Funded studies were more frequently published in higher impact journals and showed greater adherence to the CONSORT checklist, but these characteristics did not translate into a greater risk of bias.

## Resumo

**Objetivo:**

Verificar a associação entre a presença de conflito de interesses e resultados pró-financiadores em ensaios clínicos randomizados (ECRs) de cirurgia da mão. Como objetivo secundário, avaliar a influência do financiamento da indústria sobre os dados bibliométricos desses ECRs.

**Métodos:**

Examinamos 178 ECRs publicados entre 2013 e 2023 em 5 periódicos de alto impacto com foco na mão, punho, cotovelo ou nervos periféricos, nas bases de dados MEDLINE, Embase e Scopus. Dos estudos incluídos, avaliamos a presença de conflito de interesses declarado e analisamos sua associação com o desfecho primário (favorável vs. não favorável). Avaliamos adicionalmente a associação com outras variáveis, incluindo conformidade com as diretrizes Padrões Consolidados de Relatórios de Ensaios (CONSORT, do inglês Consolidated Standards of Reporting Trials), número de autores e fator de impacto dos periódicos. O risco de viés dos estudos incluídos foi avaliado utilizando a ferramenta Risk of Bias 2 (RoB 2). Todas as avaliações foram realizadas por dois revisores independentes, seguindo protocolo previamente definido. Esta revisão sistemática não foi previamente registrada em plataformas públicas, como a International Prospective Register of Systematic Reviews (PROSPERO).

**Resultados:**

Não encontramos relação entre presença de conflito de interesses e resultados pró-financiadores (35,4% vs. 65,6%; *p* = 0,503). Estudos financiados pela indústria foram publicados em periódicos com fatores de impacto significativamente maiores do que estudos não financiados pela indústria (3,05 vs. 2,2) ( *p* < 0,001), com diferença significativa no número de autores financiados (1,84 vs. 0,07) ( *p* < 0,001).

**Conclusão:**

Não foi observada associação significativa entre o tipo de financiamento e a obtenção de resultados favoráveis em ensaios clínicos randomizados na cirurgia da mão. Estudos financiados foram mais frequentemente publicados em periódicos de maior impacto e apresentaram maior adesão ao *checklist* CONSORT, mas essas características não se traduziram em maior risco de viés.

## Introduction

Quality clinical research requires, among other things, financial and industry support to make its products real. A conflict of interests (COI) is defined as “a set of conditions in which professional judgment concerning a primary interest (such as a patient's wellbeing or the validity of research) tends to be influenced by a secondary interest (e.g., financial gain)”. Thus, the target audience for academic articles should be aware of the scope of industry influence. 1 2

The financial COI includes, but is not limited to, grants/support for research, acting as an advisor, consultant or public defender, indirect financial support (e.g. travel reimbursement, equipment), stock ownership, personal fees, direct employment, lecture fees, writing or review of the topic discussed in the manuscript, speakers or board members, royalties, and patents (planned, pending or issued), regardless of the amount involved. Non-financial COIs may include, but are not limited to, personal, institutional, and professional opinions as well as relationships that may impair scientific objectivity. 3

One of the main concerns in COI research is that the company funding a trial may influence its results. The Consolidated Test Reporting Standards (CONSORT) guidelines require disclosure of study funding. The International Committee of Medical Journal Editors (ICMJE) also recommends disclosing financial ties between authors and sponsors. While the presence of COI or funding disclosures does not preclude consideration of an article for publication, the editorial team reserves the right to decline an article if it considers the scientific method flawed or the funder has compromised the study's integrity. 3 4

Previous studies 1 2 5 6 have found an association between industry sponsorship and a greater likelihood of reporting favorable efficacy results and conclusions, higher rates of disagreement between study results and conclusions compared to non-industry-funded studies, in addition to suppression of negative results. 2 5 6 7

Several studies have reported their results on the subject in medical and non-medical specialties (medical clinic, general surgery, plastic surgery, orthopedics, and physiotherapy). 1 3 8 9 10 11 12 Although they have explored this relationship in other specialties, research in hand surgery lacks sufficient research on how industry funding and COI affect the reported results and conclusions.

In the presumed COI declaration, the hypothesis that there is a relationship between the explicit COI declaration and positive outcomes (primary, secondary, favoring the device) is justified. The primary objective of the present study was to verify the association between the presence of COI with pro-funder results in randomized controlled trials (RCTs) in hand surgery.

## Methods

The present systematic review was prepared in accordance with the Preferred Reporting Items for Systematic reviews and Meta-Analyses (PRISMA) guidelines. 13 This is a secondary study, conducted independently by researchers linked to reference centers in hand surgery. To ensure the impartiality of data selection and analysis, no institutional identification was used at any stage of the methodological process.

### Search strategy and article selection

The search was conducted in the MEDLINE database via PubMed, Embase, and Scopus, following protocols similar to those of previous studies. 1 6 12 We used the descriptors *surgery of the hand* OR *hand surgery* OR *surgery of the hands* OR *hand's surgery* OR *surgeries of the hand* . Only articles published in English, Portuguese, or Spanish were included, as these are the most prevalent languages in the databases consulted and in the reviewers' domain, ensuring accurate data extraction and analysis. The searches were conducted between March 1 and 31, 2024. The articles were independently selected by two reviewers. Five important surgical journals were chosen based on the impact factor, international indexation, and academic recognition within the hand surgery community: *The Journal of Bone & Joint Surgery* (FI 5.3); *Journal of Hand Surgery* (FI 2.3); *The Journal of Hand Surgery* (European Volume) (FI 1.8); *HAND* (1.8); and *Revista Brasileira de Ortopedia* (FI 1.2).

The titles and abstracts of the articles identified were selected to exclude those clearly irrelevant to the focus of the review. The articles selected in the initial title/abstract screening were analyzed.

### Inclusion/exclusion criteria

Randomized controlled trials, published between 2013 and 2023, peer-reviewed, with themes related to hand, wrist, elbow, and peripheral nerve surgery (hereinafter collectively referred to as “hand articles”) were included. Editorials, letters to the editor, comments, errata, and other types of non-scientific publications were excluded. The criteria adopted for defining an RCT included: participation of living humans in the study; analysis of a health care-related intervention; presence of a comparator group; and allocation of participants through randomization.

In cases in which the primary outcome was not explicitly defined, favoritism was determined by two independent reviewers based on the results and conclusions sections. In case of disagreement, the final decision was made by a third reviewer with methodological experience, by consensus.

### Specific outcomes

The articles were evaluated for adherence to the CONSORT guidelines, declared COI among authors, the number of authors (including those funded), sample size, and source of study support.

Funding for each study was categorized as industry-funded, not industry-funded (other funding), or unfunded. A study was considered industry-funded if at least one author was listed as an employee of a healthcare industry, or if financial support from a pharmaceutical company was recognized. A study was considered non-industry-funded if it was funded by a non-industry entity (e.g., a government agency or academic institution). Studies that did not receive any funding were considered unfunded. Studies that received funding from industry or other non-industrial sources were categorized, in both cases, as studies with some type of funding.

Narrative outcomes and conclusions were designated as *favorable* or *unfavorable* . In the results section, a favorable primary/secondary outcome was attributed to RCTs that showed statistically significant positive results. An outcome was considered *unfavorable* when negative results were reported. In evaluating the conclusion sections, the attribute *favorable* was used when the authors declared or suggested favorability toward the target intervention. An outcome was deemed *unfavorable* when the authors declared or suggested favorability in relation to the comparison or control group. In cases in which there was conflict over whether to favor the intervention, a third evaluator determined the outcome.

## Risk of bias analysis

The risk of bias of the included studies was assessed based on the Risk of Bias 2 (RoB 2) tool, proposed by the Cochrane Collaboration, which includes 5 domains: (1) bias resulting from the randomization process; (2) bias due to deviations from the intended intervention; (3) bias due to missing data; (4) bias in the measurement of the outcome; and (5) bias in the selective reporting of results.

Each domain was classified as low risk of bias, some concern, or high risk of bias, generating an overall judgment for each study. The assessment was conducted using the methodological information and results described in the articles.

### Interrater agreement

The categorization of studies regarding the favorability of outcomes, the presence of COI, adherence to the CONSORT checklist, and the judgment of the risk of methodological bias (RoB 2) was performed independently by two reviewers with methodological training. For all these subjective variables, the agreement between the judgments was evaluated using Cohen's Kappa coefficient, considering the categories assigned to each study, with values below 0.40 indicating weak agreement; between 0.40 and 0.59, moderate; between 0.60 and 0.79, substantial; and ≥ 0.80, almost perfect.

The results were summarized in tables and used in stratified analyses to explore potential associations between risk of bias and type of funding.

## Statistical plan

The analysis of variance (ANOVA) test was used to analyze the relationship between funding, authorship, and quantitative variables, including the number of authors, the journal impact factor, and the sample size. The Chi-squared test was used to analyze the relationships between funding, CONSORT adherence, COI declaration, primary and secondary outcomes, qualitative conclusion (favorable or unfavorable to the intervention), and global classification of risk of bias (RoB 2).

Statistical analyses were performed using the IBM SPSS Statistics for Windows (IBM Corp.) Version 26.0 (2019), Minitab 21.2 (Minitab, LLC) (2022), and Microsoft Excel Office (Microsoft Corp.) 2010 software. A statistical significance level of *p* < 0.05 was adopted.

## Results

**Fig. 1:**
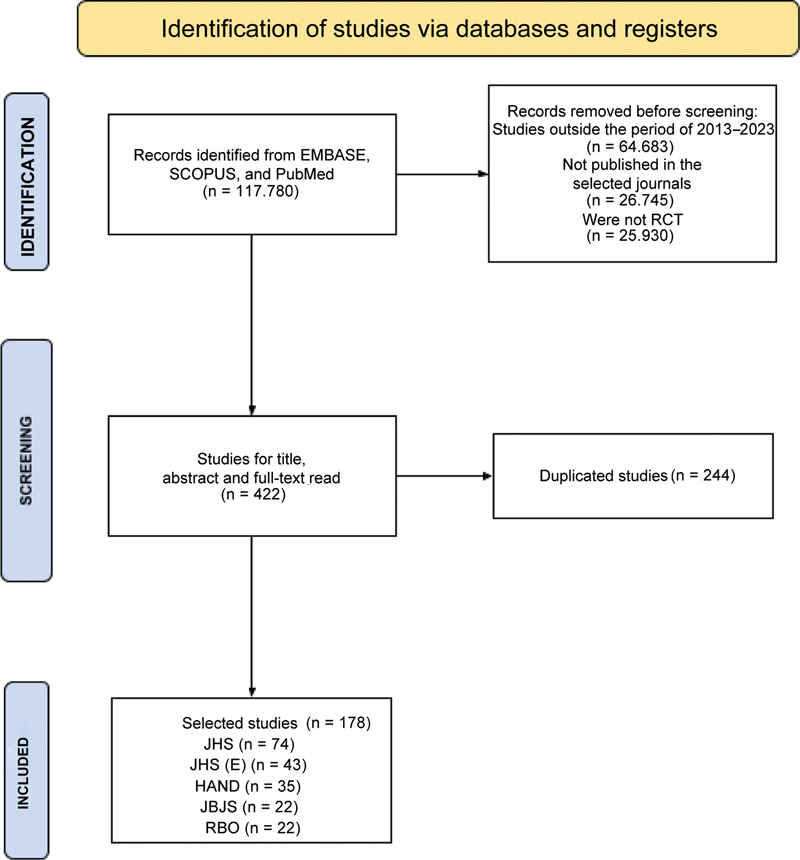
Flowchart according to Preferred Reporting Items for Systematic reviews and Meta-Analyses (PRISMA) protocol for study selection. **Abbreviations:** JHS, *Journal of Hand Surgery* ; JHSE, *Journal of Hand Surgery* (European Volume); JBJS, *The Journal of Bone & Joint Surgery* ; RBO, *Revista Brasileira de Ortopedia* ; RCT, randomized clinical trial.

At the end of the research, of the 117,780 studies found, 178 RCTs that met the inclusion criteria were included ( Fig. 1 ).

We found no relationship between the presence of COI and pro-funder results, whether primary (35.4% vs. 65.6%) or secondary (68.5% vs. 31.5%), favorable or not to the test device (68.6% vs. 31.4%) ( Tables 1 2 3 ).

**Table 1:** Relationship of favoring the primary outcome with the presence of funding and conflicts of interest

		Unfavorable		Favorable		*p* -value
		N	%	N	%	
**All**	**No COI declaration**	72	65.5%	43	63.2%	0.764
	**With COI declaration**	38	34.5%	25	36.8%	
**Other funding**	**No COI declaration**	8	27.6%	5	33.3%	0.692
	**With COI declaration**	21	72.4%	10	66.7%	
**Industry-funded**	**No COI declaration**	2	11.8%	0	0%	0.274
	**With COI declaration**	15	88.2%	15	100%	
**Some funding**	**No COI declaration**	10	21.7%	5	16.7%	0.587
	**With COI declaration**	36	78.3%	25	83,3%	
**Unfunded**	**No COI declaration**	62	96.9%	38	100%	0.271
	**With COI declaration**	2	3.1%	0	0%	

**Abbreviation:** COI, conflict of interests.

**Table 2:** Relationship of favoring the secondary outcome with the presence of funding and conflicts of interest

		Unfavorable		Favorable		*p* -value
		N	%	N	%	
**All**	**No COI declaration**	85	68.5%	29	54.7%	0.078
	**With COI declaration**	39	31.5%	24	45.3%	
**Other funding**	**No COI declaration**	12	36.4%	1	9.1%	0.086
	**With COI declaration**	21	63.6%	10	90.9%	
**Industry-funded**	**No COI declaration**	1	5.9%	1	6.7%	0.514
	**With COI declaration**	16	94.1%	14	93.3%	
**Some funding**	**No COI declaration**	13	26.0%	2	7.7%	0.057
	**With COI declaration**	37	74.0%	24	92.3%	
**Unfunded**	**No COI declaration**	72	97.3%	27	100%	0.388
	**With COI declaration**	2	2.7%	0	0%	

**Abbreviation:** COI, conflict of interests.

**Table 3:** Relationship of the conclusion favorable to the intervention with the presence of funding and conflicts of interest

		Unfavorable		Favorable		*p* -value
		N	%	N	%	
**All**	**No COI declaration**	59	68.6%	56	60.9%	0.281
	**With COI declaration**	27	31.4%	36	39.1%	
**Other funding**	**No COI declaration**	7	30.4%	6	28.6%	0.892
	**With COI declaration**	16	69.6%	15	71.4%	
**Industry-funded**	**No COI declaration**	1	9.1%	1	4.8%	0.466
	**With COI declaration**	10	90.9%	20	95.2%	
**Some funding**	**No COI declaration**	8	23.5%	7	16.7%	0.455
	**With COI declaration**	26	76.5%	35	83.3%	
**Unfunded**	**No COI declaration**	51	98.1%	49	98.0%	0.978

**Abbreviation:** COI, conflict of interests.

In the assessment of the baseline bibliometric variables, statistical significance was demonstrated for the number of funded authors (mean of 1.34 for other funding, 1.84 for industry, and 0.07 for unfunded) and the journal's impact factor (2.5 vs. 3.05 vs. 2.2) ( Table 4 ).

**Table 4:** Bibliometric data of the studies in relation to the type of funding of the studies

Bibliometric data	Funding	N	Mean	Median	Standard deviation	95% CI	*p* -value
**Number of Authors**	Other funding	44	4.93	5	1.35	4.53-5.33	0.241
	Industry	32	5.53	6	2.08	4.81-6.25	
	Unfunded	102	5.00	5	1.68	4.67-5.33	
**Number of funded authors**	Other funding	44	1.34	0	2.10	0.72-1.96	< 0.001
	Industry	32	1.84	1	2.26	1.06-2.62	
	Unfunded	102	0.07	0	0.38	0–0.14	
**Impact factor**	Other funding	44	2.50	2,3	1.15	2.16-2.84	0.001
	Industry	32	3.05	2,3	1.45	2.55-3.55	
	Unfunded	102	2.20	1,8	0.89	2.03-2.37	
**Sample size**	Other funding	44	88.43	64,5	76.67	65.78-111.08	0.627
	Industry	32	83.31	58,5	63.71	61.24-105.38	
	Unfunded	102	78.30	65	47.67	69.05-87.55	

**Abbreviation:** COI, conflict of interests.

Comparing the three groups, the analysis of the qualitative variables *CONSORT Adherence* and *COI declaration* showed statistical significance ( Table 5 ). Industry-funded studies showed a CONSORT adherence rate of 56.3% compared to other funded studies (50%) and unfunded studies (28.4%) ( *p* = 0.004). The positive rate of *COI declaration* was 93.8%, 70.5%, and 2.0%, respectively, in the respective groups ( *p* < 0.001).

**Table 5:** Characteristic of the studies regarding the type of funding

Characteristic of the studies		Other funding		Industry		Unfunded		Total		*p* -value
		N	%	N	%	N	%	N	%	
**CONSORT adherence**	No	22	50.0%	14	43.8%	73	71.6%	109	61.2%	0.004
	Yes	22	50.0%	18	56.3%	29	28.4%	69	38.8%	
**COI declaration**	No	13	29.5%	2	6.3%	100	98.0%	115	64.6%	< 0.001
	Yes	31	70.5%	30	93.8%	2	2.0%	63	35.4%	
**Primary outcome**	Unfavorable	29	65.9%	17	53.1%	64	62.7%	110	61.8%	0.503
	Favorable	15	34.1%	15	46.9%	38	37.3%	68	38.2%	
**Secondary outcome**	Unfavorable	33	75.0%	17	53.1%	75	73.5%	125	70.2%	0.064
	Favorable	11	25.0%	15	46.9%	27	26.5%	53	29.8%	
**Favorable to the device**	Unfavorable	23	52.3%	11	34.4%	52	51.0%	86	48.3%	0.217
	Favorable	21	47.7%	21	65.6%	50	49.0%	92	51.7%	

**Abbreviations:** COI, conflict of interests; CONSORT, Consolidated Standards of Reporting Trials.

The use of the RoB 2 tool enabled assessment of the risk of bias in the included studies ( Table 6 ). Given the large number of studies included, it was decided to present the results in an aggregate manner. Detailed assessment data by study and domain are available as described in the data availability section. The interrater agreement coefficient (Cohen's Kappa) of 0.79 suggests substantial agreement. Most studies were classified as low risk of bias (90/178; 50.6%), followed by some concern (73/178; 41.0%) and high risk (15/178; 8.4%). When stratified by type of funding, a similar distribution was observed across groups: industry-funded studies showed 50% low risk, 40.6% some concern, and 9.4% high risk. Statistical analysis did not demonstrate a significant association between funding and risk of bias ( *p* = 0.537 for unfunded; *p* = 0.996 for *some funding* ).

**Table 6:** Risk of bias (RoB 2) stratified by funding category in included studies (n = 178)

Type of funding	Risk of bias analysis (Rob 2)				
	Low risk	Some concern	High risk	Total	*p* -value
Unfunded	51	43	8	102	0.537 a
Industry-funded	16	13	3	32	
Other funding	24	16	4	44	
**Some funding**	**39**	**30**	**7**	**76**	0.96 a
**All**	**90**	**73**	**15**	**178**	**0.79** b

**Notes:**^a^ Pearson's Chi-squared test. ^b^ Interrater agreement coefficient (Cohen's Kappa) = 0.79.

## Discussion

In our sample, industry-funded studies were, on average, published in journals with a higher impact factor (3.05; 95% CI 2.55–3.55) than unfunded studies (mean 2.2; 95% CI 2.03–2.37), indicating a statistically significant difference ( *p* < 0.001). There was no statistically significant difference between studies with other funding (mean 2.5; 95% CI 2.16–2.84) compared with the other groups.

Several authors have already warned about the potential influence of industry on the scientific process, not only in the results but also in editorial interests and in a greater propensity to publish findings favorable to the devices under study. 6 14 15 16 17

However, regarding the positivity assessment of the primary outcomes between the unfunded and funded groups ( *p* = 0.503) in our study, it was not possible to rule out the hypothesis that both groups would have similar positivity proportions. In this context, the primary outcome was positive in 46.9% of industry-funded studies and 37.3% of unfunded studies, with no statistically significant difference. The same occurred for the secondary outcome ( *p* = 0.064) and device-favorable outcome ( *p* = 0.217).

These findings contrast with previous literature, as demonstrated by Bhandari et al. 6 who identified a higher proportion of statistically significant pro-funder results in funded surgical RCTs.

Similar results were also reported in areas such as neurosurgery, 18 dermatology 19 , and spine surgery. 14 In the latter study, Munsch et al. 14 , for example, reported a higher proportion of positive outcomes in industry-funded studies, including among subgroups funded by public agencies, in addition to identifying bias in the selective publication of studies with negative results.

In plastic surgery, a balance was observed between funded and unfunded studies in the reconstructive area, but a greater proportion of industrial funding in cosmetic surgery. 12 This pattern is close to the profile observed in our study, focused on hand surgery, a mostly reconstructive area.

In orthopedics, Nesello et al. 20 also found a higher proportion of positive results in industry-funded studies using platelet-rich plasma, particularly in lower-level evidence studies. In contrast, our study found no significant difference between the groups in sample size, although the number of authors was higher in studies with funding ( *p* < 0.05).

Regarding the quality of reporting, we observed greater adherence to the CONSORT checklist among industry-funded studies (56.3%), followed by other funding (50%) and unfunded studies (28.4%), a statistically significant difference ( *p* = 0.004). Adherence to CONSORT reinforces the transparency and reproducibility of studies and, although not mandatory, its adoption is seen as indicative of methodological rigor. The CONSORT checklist presents 25 items that assist in the transparency, reproducibility, and proper interpretation of the study results. 21 22 23 24

However, our study also has relevant limitations. The selection bias of the journals, all high-impact, may have favored the inclusion of RCTs with greater methodological rigor, reducing the heterogeneity of the sample. In addition, by limiting the selection to the post-2013 period, there may have been a higher prevalence of studies that were subject to stricter editorial transparency requirements.

Another critical point concerns the reliability of COI declarations. Studies such as Hannon et al. 25 and Tian et al. 26 identified significant discrepancies between copyright statements and records in public databases, such as open payments. This type of understatement can mask the actual extent of funding and impact the categorization of studies.

Finally, when assessing the risk of methodological bias (RoB 2), we identified that most studies were classified as low risk, with a relatively homogeneous distribution across funding groups. Statistical analysis did not indicate a significant association between the type of funding and the overall judgment of the risk of bias ( *p* ≥ 0.05). Still, the presence of studies with *some concern* in all categories indicates that attention to methodological quality should be constant, regardless of the source of funding.

However, it is worth noting that RoB 2 may not fully capture structural biases associated with funding, such as comparator selection, outcome setting, or selective reporting. As pointed out by Lundh et al. 27 and reinforced by Devji et al., 5 industry-funded studies may present more favorable conclusions to the sponsor, even when classified as low risk of bias, thereby requiring caution in interpreting these results.

Thus, although funding should not be used in isolation as a criterion for judging a study's validity, it should be considered in the context of its reporting, methodological transparency, and its potential to influence interpretations. Our study contributes by demonstrating that funded studies, when well-designed and published in journals with high editorial rigor, do not necessarily present a greater risk of bias, but still require careful critical analysis.

### Study limitations

The selection bias of journals is relevant: the sample was composed exclusively of high-impact journals, which may have excluded relevant RCTs published in less visible journals, potentially biasing the analysis in favor of studies with greater methodological rigor and a higher frequency of declared funding.

In addition, the categorization of funding and COI was based solely on the authors' statements, which may be subject to omissions or inaccuracies—as demonstrated by studies comparing these statements with public records of industry payments.

Finally, the absence of prior registration of the protocol on public platforms, such as International Prospective Register of Systematic Reviews (PROSPERO), constitutes a limitation of the study.

## Conclusion

The present study did not identify robust evidence that the type of funding—whether from industry, other sources, or no stated funding—is associated with a higher proportion of favorable intervention outcomes in randomized clinical trials in hand surgery. It was observed that the funded studies were, on average, published in higher-impact journals and showed greater adherence to the CONSORT checklist, which may reflect greater methodological rigor or stricter editorial requirements.

Despite these findings, caution is recommended in interpretation, as the analysis was not adjusted for potential confounders, and tools such as RoB 2 may not fully capture structural biases related to funding, outcome definition, and selective reporting. In addition, the inclusion of high impact factor journals may have limited the methodological variability of the studies evaluated.

In summary, although funding has not been directly associated with greater methodological risk or with a trend toward favorable results, the potential influence of funders on the design, conduct, and reporting of studies remains a valid concern and should continue to be subject to critical scrutiny in future systematic reviews and editorial practices.
